# Development, Validation, and Implementation of an Innovative Mobile App for Alcohol Dependence Management: Protocol for the SIDEAL Trial

**DOI:** 10.2196/resprot.5002

**Published:** 2016-02-17

**Authors:** Pablo Barrio, Lluisa Ortega, Xavier Bona, Antoni Gual

**Affiliations:** ^1^ Addictions Unit Clinical Neuroscience Institute Hospital Clinic of Barcelona Barcelona Spain; ^2^ Pulso Ediciones Patient Support Programs Barcelona Spain

**Keywords:** alcohol dependence, alcoholism, telemedicine, mobile applications, eHealth, adherence, patient compliance, consumption register

## Abstract

**Background:**

Information and communication technologies (ICT) have become one of the main pathways to the new paradigm of increased self-management of chronic conditions such as alcohol dependence. Validation of some mobile phone apps has begun, while validation of many others is forthcoming.

**Objective:**

To describe the protocol for validation of a new app called SIDEAL (an acronym of the Spanish name “Soporte Innovador a la persona con DEpendencia del ALcohol,” or innovative support for people with alcohol dependence).

**Methods:**

The project consists of 3 complementary, consecutive studies, including a pilot feasibility study, a qualitative study using focus groups, and, finally, a randomized controlled trial where patients will be randomized to standard treatment or standard treatment plus SIDEAL. During the pilot study, feasibility, usability, and acceptance by users will be the main outcomes explored. An electronic questionnaire will be sent to patients asking for their opinions. Focus groups will be the next step, after which improvements and refinements will be implemented in the app. During the final phase, consumption variables (heavy drinking days per month, mean standard drinks per day) will be investigated, in order to test app efficacy.

**Results:**

Because of the encouraging results with previous similar apps, we expect patients to widely accept and incorporate SIDEAL into their therapeutic options. Significant reductions in drinking-related variables are also expected. The pilot study has concluded with the inclusion of 29 patients. Results are expected to be available soon (expected mid-2016).

**Conclusions:**

SIDEAL may represent a useful, reliable, effective, and efficient tool to complement therapeutic options available to both patients and professionals.

## Introduction

According to the World Health Organization [1], alcohol is the third largest contributor to the global burden of disease in developed countries, accounting for massive costs at both the individual and society level. Alcohol dependence, now formally labeled severe alcohol use disorder, is a medical, chronic, relapsing disease. In Spain, for example, it accounts for nearly 75% of the total burden of disease attributable to alcohol [2]. Because of its chronic, relapsing-remitting nature and high costs, there is a strong need to develop new ways to provide continuous care.

Information and communication technologies (ICT) could offer an approach to the addiction field through a new paradigm of increased self-management. Moreover, technology has the potential to provide continuous personalized care at a fraction of the cost of standard care. Existing evidence supports the suitability of chronic diseases as a target for technological assistance, to improve quality of life and reduce health care costs [3]. These facts have not gone unnoticed by national and international institutions, which are now clearly supporting and encouraging the development of such technologies. A clear example is the Horizon 2020 initiative of the European Commission, with an approximate budget for ICT-related projects of €560 million.

Intelligent mobile phones, or *smartphones* are currently widely used in Spain (4). Besides the usual telephone service, they offer computer-like functions with advanced software capabilities. Their portability, ease-of-use, and ubiquity make them excellent tools to enhance the management of chronic conditions. A new horizon of interactions between health care services and users with mobile phones as mediators can be envisioned. The range of possibilities includes scheduling visits for psychoeducation, monitoring, brief interventions, and biometric assessments.

A multitude of mobile apps specifically focusing on alcohol are currently available in the software market. However, recent reviews point out that a majority of them encourage drinking, while a smaller number support the reduction of problematic drinking. However, little research supporting their use is available [4,5]. One randomized, controlled trial was conducted, and encouraging results were reported [6]. Additional trials are expected in the near future, in a growing effort to apply scientific standards to this continuously and rapidly changing new area of ICT.

Recent publications [4,7-9] identified two key elements critical to the efficacy of apps in the management of chronic diseases. These include ongoing monitoring and use of push technology (which provides sustained proactivity). Other success-conditioning factors are usability, institutional support, expert endorsement, and individualized adjustment or tailoring. It is also strongly urged that these apps be evaluated using the scientific method, which will allow for the implementation of apps based not only on their usability, but also on their validity, effectiveness, and efficiency.

Mobile phones have been suggested to be suitable platforms for the management of alcohol use disorders via apps that integrate different functions. These include skills training, motivation enhancement, psychoeducation, social support, relapse prevention, and reminders (medical visits, medication schedules, health events). In addition, technology allows for the implementation of a system for continuous monitoring and ecological momentary assessment ("in the moment") [4]. The information gathered can then be displayed to the user (thus providing feedback) in an attractive way, facilitating modulation of the problematic behavior. All these features and functions therefore allow not only for simple and efficient collection of information, but also for the combination of this information with other functions to potentially encourage motivation, decrease problems associated with consumption, and even support relapse prevention [10].

The SIDEAL project (an acronym of the Spanish name “*Soporte Innovador a la persona con DEpendencia del ALcohol*,” or innovative support for people with alcohol dependence) was undertaken in this context. SIDEAL is a public-private collaboration of Lundbeck Spain SA, Pulso Ediciones, SL, the Subdirectorate of Drug Addictions of the Catalonia regional government, and the Addictions Unit of the Clinic Hospital of Barcelona. A Web-based app system has been developed to support alcohol-dependent patients. This paper reports its theoretical basis and development process, as well as the protocols of the different studies that will be conducted to validate its usability and efficacy.

## Methods

### Theoretical Basis and Patient Implementation

The main source of theoretical guidance for SIDEAL comes from motivational interviewing (MI) principles [11]. MI is intended to help people move through the stages of change [12] in a nonjudgmental, facilitative manner. Although it has a directive, goal-oriented style, it is respectful of patients’ goals. MI also encourages an individual, tailored approach for each patient. Therefore, SIDEAL is respectful of patients’ goals, and allows for tailoring of the app according to their preferences. Other principles adopted in the development of the app include design simplicity, noninvasive proactivity, privacy, professional endorsement and support, and adjustment to individual needs. An increasing number of guidelines and recommendations for developing apps aimed at behavioral change [13,14] are also currently available and continue to be useful as a source of practical guidance.

While other apps currently being validated, such as the Location-Based Monitoring and Intervention for Alcohol Use Disorders (LBMI-A) [8], have been designed as stand-alone, self-administered, independent treatments for alcohol patients, the purpose of SIDEAL is to become a tool that both patients and professionals can incorporate into their therapeutic armamentarium. Although the app may work as a stand-alone option, we think that it is best used under the principles of shared information and decision-making between patients and professionals. All information produced by users is transferred to a Web system to which professionals have access. In order to respect patients’ privacy, however, users always have to proactively allow the information to be transferred to the Web by clicking a specific button in the app.

The app is currently available free of charge in the main app markets (ie, Apple Store and Google Play). However, to create a user account and tailor and personalize features, patients should have an initial visit with a professional, during which both the patient and professional will configure the user’s account via the Web system. For example, the main treatment goal will be set (choosing between complete abstinence and reduction). If reduction is selected, a desired daily consumption limit will also be set. If a patient is taking any specific medication, this will also be recorded in the user’s account. Based on patient preferences, professionals will be able to select which psychoeducational text message (short message service, SMS) the user will receive on a weekly basis.

### SIDEAL Development Process

The development and testing phases of the Web-based app system were conducted from September 2013 to December 2014 by a collaborative team of specialists in addictions (psychiatrists) and software engineers, as well as graphic designers and project managers, with previous experience in the development of patient support software. All user requirements, functions, and software design were first established, and the installation (soft and hard), operational key functions, and performance requirements were subsequently tested to evaluate the entire system.

In addition to compliance with security and privacy rules, relevant app requirements and functionalities include easy-to-use functions for selecting a program (reduction or abstinence); recording daily alcohol consumption (standard units); graphically monitoring standard units in reference to the established limit; accessing educational information; and displaying a telephone help line (public or private). For users monitored by a physician who previously created a user profile for them through the Web interface, additional app functions were included for downloading consumption goals and prescribed therapies from the dedicated professional Web interface; recording and monitoring treatment adherence; receiving automated personalized feedback (weekly via the app); receiving one general piece of advice weekly (a text message selected at random from a pool of 50); and receiving surveys for users to provide feedback.

All 50 text messages were selected and reviewed by the investigator’s panel based on best practice standards and consensus. Updated published educational information was used.

### SIDEAL Functionalities

**Figure 1:**
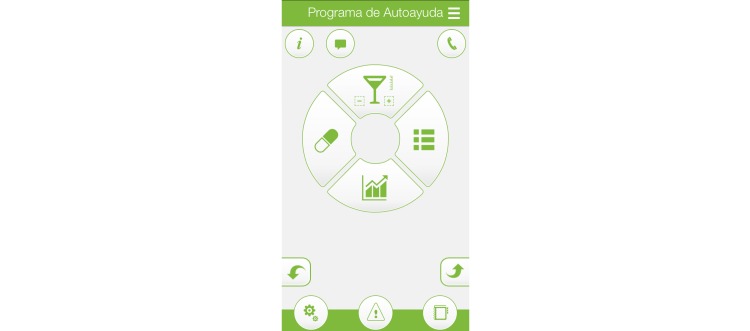
Initial screen.

The different “modules” or functionalities included in the app may be accessed in parallel, with no restrictions, in contrast to the stepwise approach implemented in other apps. Figure 1 shows the initial screen, where all functionalities may be accessed.

#### Consumption Records

**Figure 2:**
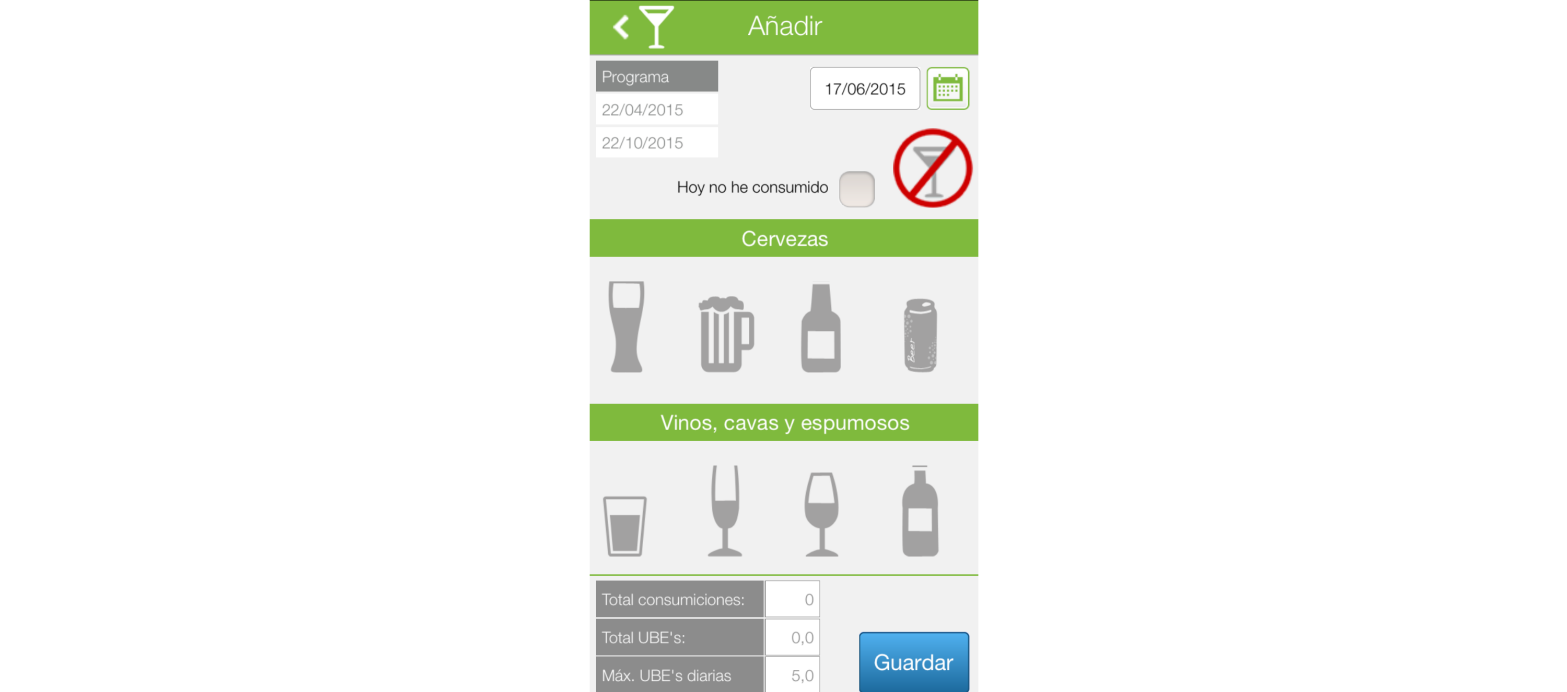
Consumption register.

Patients record alcohol consumption using icons representing alcoholic drinks. The system automatically converts this information into standard units and displays it in a graphic where the agreed limit is also displayed. The user can set reminders that will appear when consumption limits are exceeded. Consumption may be introduced gradually throughout the day, all at once, or even on subsequent days using the calendar function, which allows for the selection of previous dates. The system also allows patients to record that no consumption has occurred during the day. Figure 2 shows a consumption register screen.

#### Drug Adherence Record

**Figure 3:**
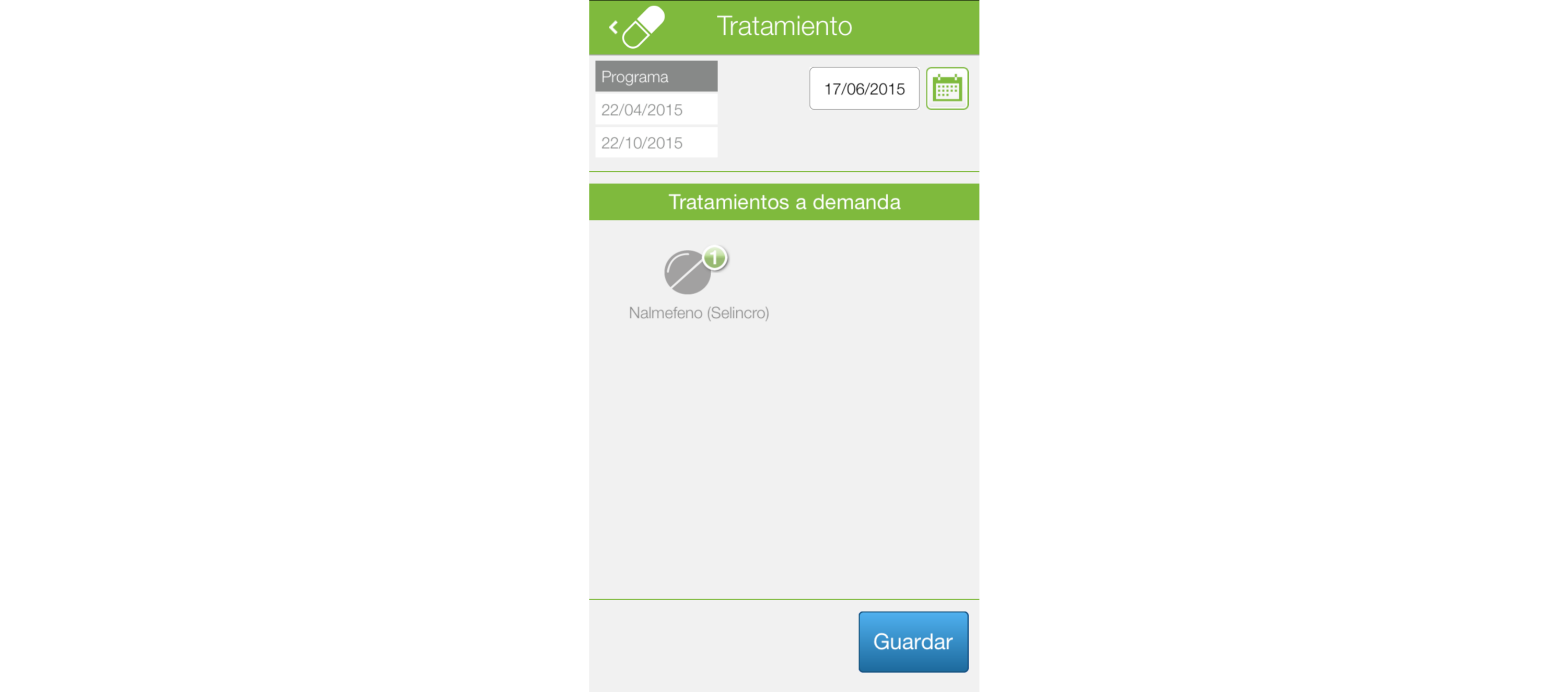
Pharmacological adherence register.

If the patient is receiving an alcohol-specific drug treatment, the system also allows for recording daily drug compliance. The system automatically computes percent drug adherence. Figure 3 shows one of the screens of this module.

#### Calendar

**Figure 4:**
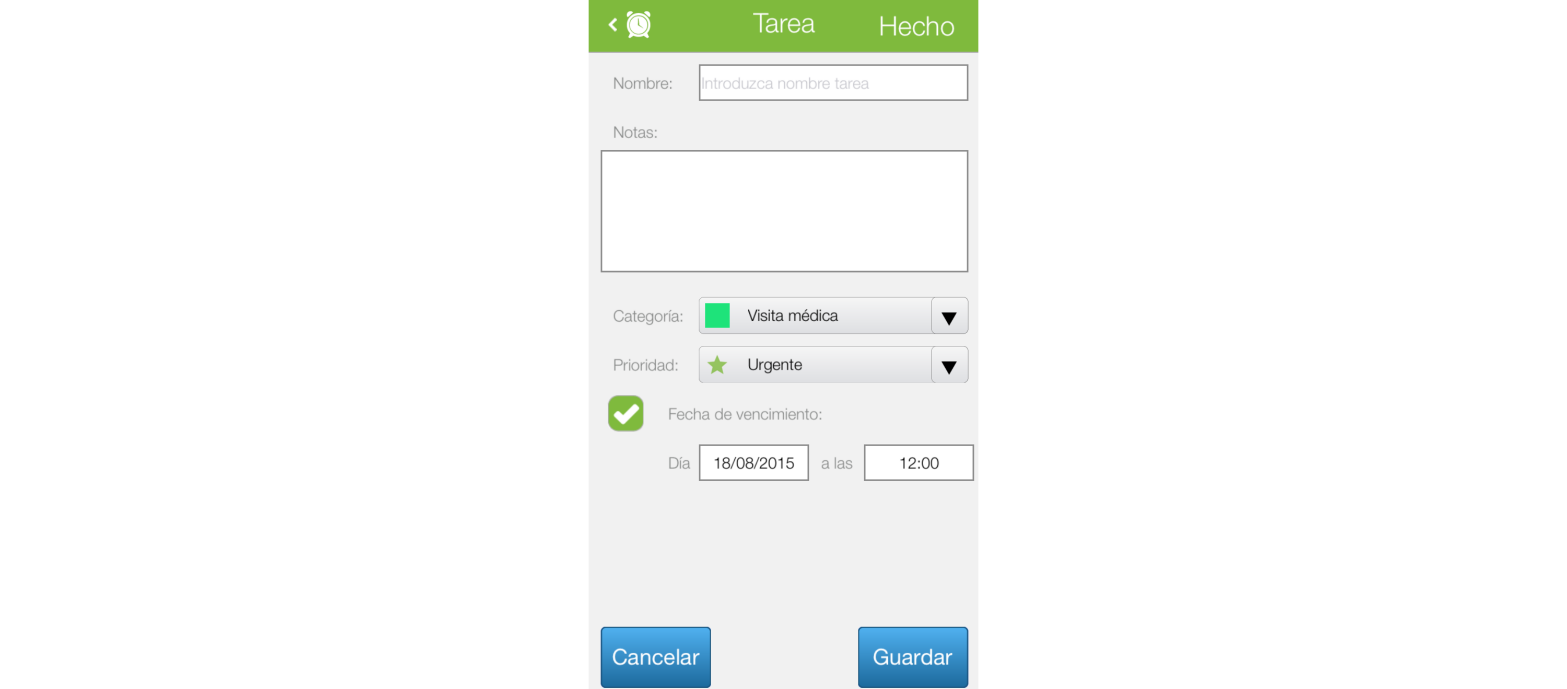
Calendar.

Patients can enter the dates of visits to different health care professionals. The system will produce reminders with push technology to remind patients of their scheduled visits. Figure 4 presents a screen showing this function.

#### Medical Information and Psychoeducation About the Disease

**Figure 5:**
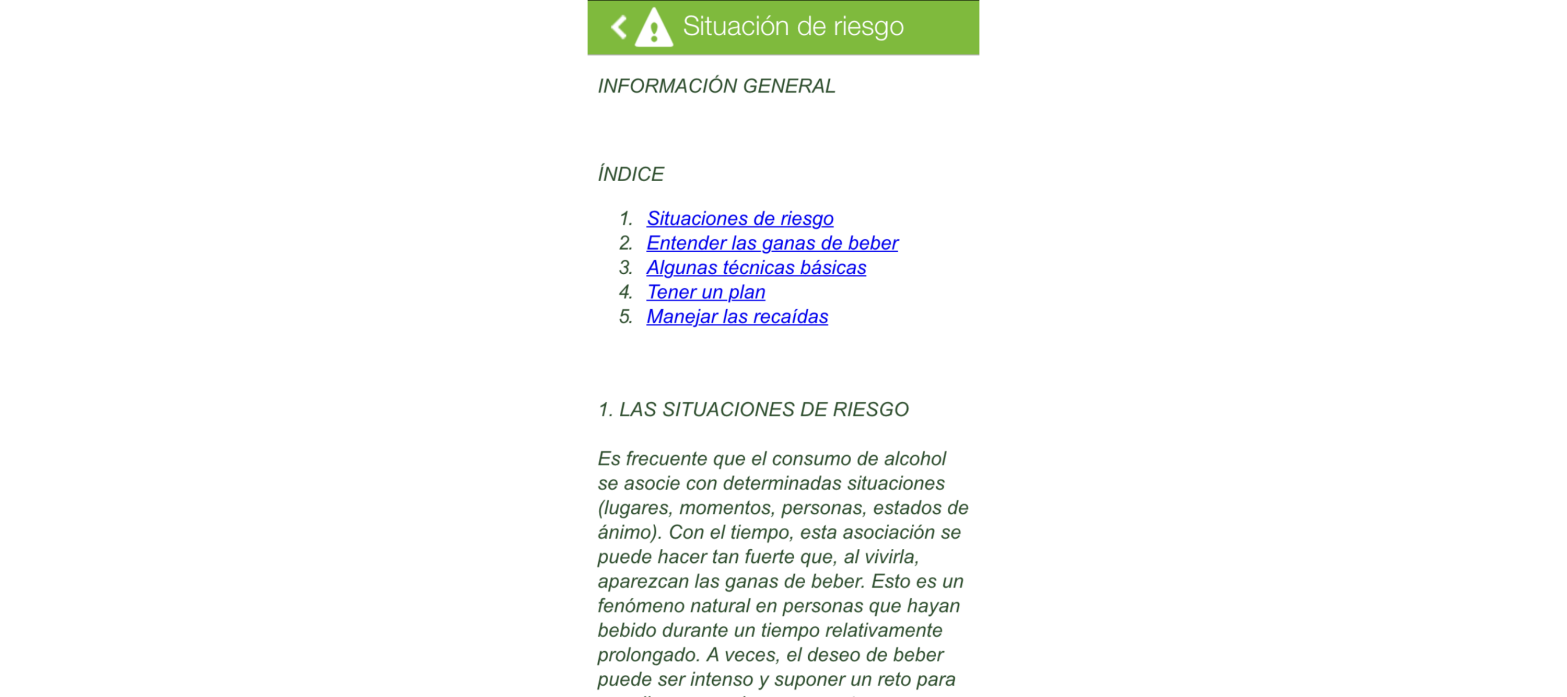
Psychoeducation module.

Basic information about alcohol use disorders is presented, as well as links to useful, trustworthy websites. A special and separate body of information on risky situations and craving is included (see Figure 5). In addition, an SMS text message with psychoeducational content is sent weekly to the user.

#### Weekly Questionnaires

Weekly surveys are sent to patients via push technology, including a questionnaire on satisfaction with their general progress, and another regarding their craving for alcohol. Both employ Likert scales and use the previous week as a reference.

#### Weekly Feedback

Once a week, via push technology, patients receive a short feedback message of a motivational nature about their use (number of days during the last week) of the consumption recording module.

### Validation Process and Methodology

We will carry out 3 successive studies to evaluate and validate SIDEAL. All studies will recruit adults (≥18 years of age) from the outpatient clinic of the Addictions Unit of the Clinic Hospital of Barcelona. Recruitment will follow a consecutive sampling strategy. Subjects will be required to sign an informed consent form before they are recruited into any study. They will also be required to have a mobile phone with an active Internet connection. Exclusion criteria for all studies will be addiction to any other substance (excluding nicotine), cognitive decline, technological illiteracy, or any other condition precluding the use of a mobile phone.

### Feasibility Study

A 6-week pilot study will first be conducted to assess SIDEAL feasibility and usability, and users’ satisfaction. As we will have equipped the app with all features suggested in the literature to maximize feasibility, usability, and satisfaction, we expect high scores on outcome variables in this first stage. In this regard, personalization, user engagement (via push technology and weekly feedback reports), reduced invasiveness and enhanced privacy, and a high-quality user interface were of critical importance in the development of the app, and are the expected drivers of the feasibility study outcomes.

Because of the pilot nature of the study, 30 patients are considered an adequate sample size. Therefore, 30 outpatients with alcohol use disorders will be given the opportunity to install the app on their mobile phone, and will be asked to use it for 6 consecutive weeks. Inclusion criteria will be adults suffering from alcohol dependence according to DSM-IV or from severe alcohol use disorder according to DSM-V (as clinically diagnosed by their psychiatrists).

At baseline, sociodemographic data (age, sex, marital status, education level) will be collected. Clinical variables related to the disease and drinking status will also be collected at baseline and at study end, and will be taken from the Timeline Follow-Back (TLFB), which will be completed for the preceding 6 weeks at both baseline and study completion. The main variables analyzed will be the number of heavy drinking days and mean alcohol consumption in units per day. When the 6-week period is over, patients will be asked to complete an electronic questionnaire on app usability and satisfaction. The questionnaire will be an adaptation of the Usefulness, Satisfaction, and Ease-of-use Questionnaire (USE Questionnaire) [15] and will consist of 30 items to be rated by users on a 7-point Likert scale (from 0 to 6). Items are grouped into 4 main categories: usefulness, ease of use, ease of learning, and satisfaction. Extra questions about which modules were found to be most useful, and a free-text section to allow patient comments on any point they consider relevant, will be added to these items. The questionnaire data will be reported according to the Checklist for Reporting Results of Internet E-Surveys (CHERRIES) checklist [16]. Complementary data regarding usability, alcohol consumption, craving modulation, and other clinical variables will also be collected from the data generated by the app.

Statistical analysis will consist of a descriptive analysis of sociodemographic and clinical variables. Acceptance and feasibility will be analyzed as percentage of days used and through the satisfaction questionnaire, for which results will be analyzed using means and standard deviations of all respondents for each category of the questionnaire. The proportions of patients reporting high, medium, or low levels of satisfaction will also be calculated. Paired *t* tests and chi-square tests will be used for analysis of drinking variables (difference from baseline to study end). These variables will be the number of heavy drinking days and the mean number of units per day. Because of the exploratory nature of the pilot study, no regression analysis will be conducted.

### Focus Groups

Two different single-session focus groups (one with patients, one with psychiatrists) will be carried out to discuss the experience with SIDEAL, and to collect suggestions and reports of errors. A maximum of 10 subjects included in the feasibility study will be selected for the focus group.

Personal interviews with some users will also be conducted to collect qualitative information. Data will be analyzed using qualitative methods. Focus group sessions will be transcribed verbatim and analyzed with NVivo 10 or a similar software package. In analyzing the transcripts, areas of consensus and discrepancy will be discussed. Coding and categorizing will follow the principles of inductive content analysis [17]. The information collected will be helpful to improve SIDEAL, a new version of which will be produced if considered necessary.

### Randomized Controlled Trial

The last step will be the completion of a randomized, controlled trial. Selected subjects will be randomized to standard treatment plus SIDEAL or to standard treatment alone. There will be no exclusion criteria with regard to what is considered standard treatment. In this sense, patients may be receiving any treatment, pharmacological or psychosocial, on an individual or group basis. The study will last 24 weeks. The initial hypothesis, based on previous research on the efficacy of alcohol apps, is that the addition of SIDEAL will improve drinking outcomes in alcohol-dependent patients, with especially significant reductions in the number of heavy drinking days and mean alcohol consumption. User acceptance and usability will be essential to achieve this objective. In addition, continuous monitoring and feedback, as well as psychoeducational content and messages, are expected to be effective for decreasing alcohol consumption.

To be included, subjects will be required to have a diagnosis of alcohol dependence or alcohol use disorder according to their psychiatrists, a drinking pattern in the previous 4 weeks consisting of >5 heavy drinking days (HDD; defined as a day with alcohol consumption ≥60 g for men and ≥40 g for women), and an average alcohol consumption above medium risk levels (ie, >40 g of alcohol/day for men and >20 g of alcohol/day for women) in the 4 weeks before study entry. Patients will be evaluated at baseline and week 24. Baseline assessments will be the same as those in the feasibility study, with the addition of the following liver enzyme tests: gamma-glutamyl transferase (GGT), aspartate aminotransferase (AST), and alanine aminotransferase (ALT); and the Addiction Severity Index (ASI) [18]. At week 24, drinking variables (number of HDDs and mean units per day) taken from the TLFB for the previous 6 weeks will be collected. Blood tests and the Addiction Severity Index will be evaluated again.

The statistical analysis will consist of a multivariate regression analysis with change from baseline to study end in number of HDDs and mean alcohol consumption per day as the dependent variables. Age, sex, baseline alcohol drinking, and ASI scores will be entered as independent variables. A secondary analysis will compare changes between groups in ASI scores and liver enzyme levels. A logistic regression analysis will also be conducted with the same independent variables, where rates of point and continuous prevalence of abstinence will be considered as the dependent variables.

For sample size calculation, using a significance level of 5%, and assuming a standard deviation for the change from baseline in number of HDD of 6 days, 45 patients in each treatment group would provide 80% power for detecting a difference between treatment groups of 4 heavy drinking days, assuming a dropout rate of 25% at week 24. Results will be reported according to the CONSORT-EHEALTH checklist [19].

## Results

The pilot study has concluded with the inclusion of 29 patients. Results are expected to be available soon.

## Discussion

Improved care, decreased cost, increased efficiency, and strengthened communication between patients and professionals are the advantages promised by mobile technology in the addiction field. A new paradigm of self-management for chronic conditions such as addiction is growing closer.

We hope that validation of this app will help patients improve the management of their condition, increasing treatment efficacy and reducing costs. There is clear support for this process from both public and private institutions, a fact that we consider essential to ensure wide and firm implementation.

We expect the app to be flexible. This means that the app will surely be improved during the validation process, and other functionalities or modules may be incorporated. Patient feedback will be indispensable for this task.

Recent studies validating similar apps for patients with alcohol disorders have reported promising results. For example, Gustafson et al [6] recently reported the results of a randomized, controlled trial of the Addiction-Comprehensive Health Enhancement Support System (A-CHESS) app, for which a significant reduction of risky drinking days was found in patients receiving the app. Dulin et al [15] reported the results of a pilot study with the app LBMI-A. Although this was an exploratory study, results were also encouraging, with patients reporting high degrees of usefulness and also showing significant reductions in hazardous alcohol use while using the system.

The complex pathophysiology of alcohol use disorders, where both psychosocial and biological factors are intertwined, should not be forgotten. In alcohol-dependent patients, many resources and treatment components are usually needed to achieve stable remission or an enduring reduction. Hence, we think that expectations of mobile technologies applied to the field of alcohol addiction should be accordingly reasonable. In this regard, we expect SIDEAL to become an add-on to the different options available to professionals and patients when devising a therapeutic plan.

A main limitation of this project is the fact that many alcohol-dependent patients may have some degree of cognitive decline [17,18], which could prevent them from using the app. Although, as previously stated, we expect the app to become a common tool for patients and therapists, there remains a low chance that patients will decrease their attendance at face-to-face visits. Other methodological concerns are the impracticality of blinding participants to study interventions and the fact that patients will be recruited from the same site, which may decrease the external validity of the study findings.

As a conclusion, we expect to add scientific knowledge and apply rigor to the ever-evolving field of mHealth. In this process, we expect SIDEAL to become a reliable, useful, and effective tool for alcohol-dependent patients and their health care professionals.

## Abbreviations

ALT: alanine aminotransferase
ASI: Addiction Severity Index
AST: aspartate aminotransferase
A-CHESS: Addiction–Comprehensive Health Enhancement Support System
CHERRIES: Checklist for Reporting Results of Internet E-Surveys
GGT: gamma-glutamyl transferase
ICT: information and communication technologies
TLFB: Time-line Follow Back
USE Questionnaire: Usefulness, Satisfaction, and Ease of use Questionnaire
